# Target-Guided Droplet Routing on MEDA Biochips Considering Shape-Dependent Velocity Models and Droplet Splitting

**DOI:** 10.3390/bios15080500

**Published:** 2025-08-03

**Authors:** Yuta Hamachiyo, Chiharu Shiro, Hiroki Nishikawa, Hiroyuki Tomiyama, Shigeru Yamashita

**Affiliations:** 1 Graduate School of Science and Engineering, Ritsumeikan University, Kusatsu 525-8577, Japan; yuta.hamachiyo@tomiyama-lab.org (Y.H.); chiharu.shiro@tomiyama-lab.org (C.S.); 2 WITZ Corporation, Nagoya 460-0004, Japan; 3 Graduate School of Information Science and Technology, The University of Osaka, Osaka 565-0871, Japan; nishikawa.hiroki@ist.osaka-u.ac.jp; 4 College of Information Science and Engineering, Ritsumeikan University, Osaka 567-8570, Japan; ger@cs.ritsumei.ac.jp

**Keywords:** digital microfluidics, biochips, MEDA, droplet routing, mathematical programming problem

## Abstract

In recent years, digital microfluidic biochips (DMFBs), based on microfluidic technology, have attracted attention as compact and flexible experimental devices. DMFBs are widely applied in biochemistry and medical fields, including point-of-care clinical diagnostics and PCR testing. Among them, micro electrode dot array (MEDA) biochips, composed of numerous microelectrodes, have overcome the limitations of conventional chips by enabling finer droplet manipulation and real-time sensing, thus significantly improving experimental efficiency. While various studies have been conducted to enhance the utilization of MEDA biochips, few have considered the shape-dependent velocity characteristics of droplets in routing. Moreover, methods that do take such characteristics into account often face significant challenges in solving time. This study proposes a fast droplet routing method for MEDA biochips that incorporates shape-dependent velocity characteristics by utilizing the distance information to the target cell. The experimental results demonstrate that the proposed method achieves approximately a 67.5% reduction in solving time compared to existing methods, without compromising solution quality.

## 1. Introduction

### 1.1. Digital Microfluidic Biochips (DMFBs)

Microfluidic technology has been widely applied in various biochemical and medical fields, including point-of-care diagnostics, enzymatic assays in clinical diagnostic systems, large-scale immunoassays, and high-throughput DNA sequencing [1,2]. Among these, Digital Microfluidic Biochips (DMFBs) have emerged as a representative example of Lab-on-Chip (LoC) systems that realize high flexibility and efficiency in experimental processes by automating the manipulation of microscopic droplets [3,4,5].

**Figure 1:**
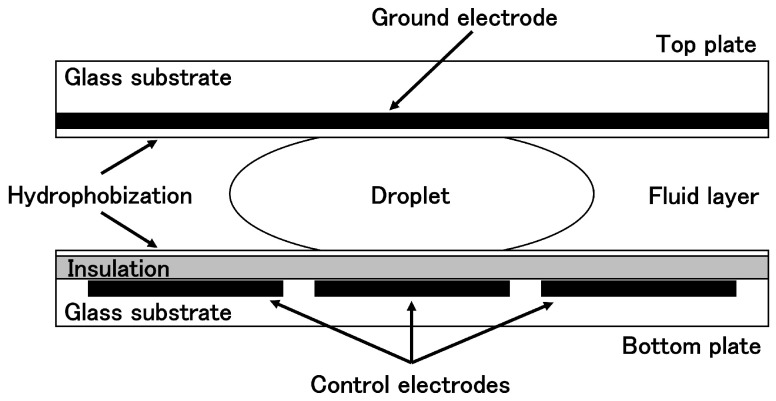
Schematic cross-section of a DMFB.

As illustrated in the Figure 1, DMFBs are structured such that a droplet is sandwiched between two electrode planes and surrounded by silicone oil or another immiscible liquid or gas. The top plate contains a continuous ground electrode, while the bottom plate features an array of individually addressable control electrodes, each slightly smaller than the droplet footprint. Both the upper and lower surfaces are hydrophobic, and the control electrodes on the bottom are electrically insulated from the liquid [6]. In this configuration, applying an electric field to only one side of the droplet creates an imbalance in interfacial tension, which in turn causes the droplet to move under the resulting force. This phenomenon is explained by the electrowetting-on-dielectric (EWOD) principle, which is based on the Young-Lippmann equation. As a result, DMFBs enable precise manipulation of nanoliter- to picoliter-scale droplets on a two-dimensional electrode array [6,7,8,9,10]. Thanks to this fine-grained control over small droplets, DMFBs serve as cutting-edge portable devices capable of executing complex experimental procedures for clinical diagnostics and biochemical analysis [11,12].

In contrast to conventional continuous-flow microfluidic biochips, which require microchannels to transport fluids and are equipped with complex structures such as micropumps and microvalves [8,9], DMFBs rely on electrical control to move droplets, resulting in a simpler architecture with higher flexibility. This makes DMFBs particularly well-suited for Point-of-Care (PoC) diagnostics in developing countries with limited resources [13]. Moreover, recent advancements in Micro-Electro-Mechanical Systems (MEMS) and microfabrication technologies have brought innovation to the design and application of DMFBs, promoting their evolution into even more compact and integrated Lab-on-Chip platforms [11,12]. As a result, DMFBs have been utilized as reliable and miniaturized platforms across a wide range of biochemical and medical applications, including DNA analysis, PCR testing, immunoassays, clinical diagnostics, and environmental toxicity assessment [8,14,15,16,17,18].

Current DMFBs offer advantages such as flexible device geometry design, easy integration with other technologies, reconfigurability, and simplified instrumentation [12]. However, as bioassays become increasingly complex and require greater concurrency, DMFBs face several functional challenges. Specifically, they are unable to control droplet volume and shape during operation, lack integrated real-time detection capabilities, and face difficulties in the fine-grained control of droplet size and volume. Additionally, they require specialized manufacturing processes and pose concerns regarding the reliability and yield of the fabricated devices [12,19,20,21]. To address these limitations, the micro-electrode-dot-array (MEDA) technology has been introduced [21,22,23].

### 1.2. Micro Electrode Dot Array (MEDA) Biochips

**Figure 2:**
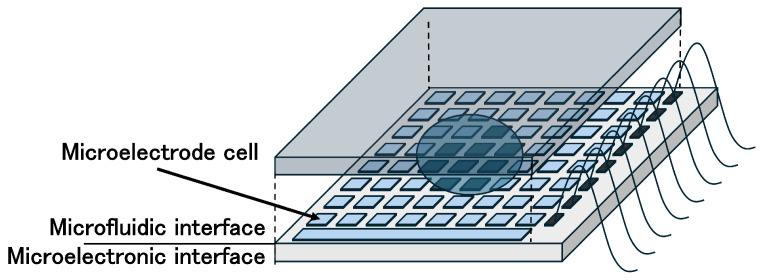
MEDA biochips.

The micro-electrode-dot-array (MEDA) biochip has been proposed as an architecture capable of overcoming the limitations of conventional DMFBs [23,24,25]. Unlike DMFBs, MEDA biochips are composed of an array of identical microfluidic units called microelectrode cells (MCs). Figure 2 shows MEDA biochips. Each MC consists of a microelectrode and a control/sensing circuit. These components can be dynamically grouped under program control to reconfigure as microfluidic modules such as mixers or diluters. MEDA-based biochips are fabricated using TSMC 0.35 µm CMOS technology, and their control circuits can operate with a 3.3 V power supply [23,25,26,27]. In this way, MEDA architecture overcomes the main challenges faced by DMFBs, such as the need for high voltages (tens of volts) and specialized fabrication processes [24].

Compared to DMFBs, MEDA biochips exhibit fundamentally different architectures and functionalities, making it impractical to directly apply existing routing and synthesis techniques. According to [21,24], the key differences are as follows:

- Unlike DMFBs, MEDA enables droplet movement in diagonal directions, significantly increasing routing flexibility and allowing faster transportation across the chip.
- By dynamically grouping multiple microelectrodes into functional units, MEDA can alter the size and aspect ratio of droplets during routing. These shape transformations affect the actuation force and resistance encountered during movement, thereby influencing droplet velocity. Consequently, routing strategies must account for these factors.
- With integrated active CMOS logic circuits, real-time sensing can be performed at any location on the chip, drastically reducing sensing response times and enabling fine-grained monitoring during assay execution.
- In MEDA, certain operations, such as lamination-based mixing, complete much faster than in DMFBs. Therefore, the previously accepted assumption that routing time is negligible no longer holds. Minimizing routing time has a significant impact on the overall performance of a bioassay.

These architectural and operational differences necessitate the development of new routing and synthesis methods tailored specifically for the MEDA platform.

### 1.3. Related Works

Numerous studies on DMFBs have been conducted from various perspectives, including design, routing, error recovery, and sample preparation [13,28,29,30]. However, as discussed in Section 1.2, the importance of routing time has increased in MEDA biochips compared to DMFBs, and it is difficult to directly apply traditional routing techniques to MEDA architectures.

In many DMFB applications, the same experiments must be repeated multiple times. For example, in drug discovery, new compounds need to be tested against a wide variety of reagents and samples, and this process is iterated frequently. In such applications, parallelism is crucial to reduce processing time, cost, and human error, and this necessitates fast and scalable routing methods [31]. The same holds true for MEDA biochips. As the number of routing iterations increases, the routing time becomes a direct bottleneck to the overall throughput of biochemical assays. Therefore, reducing the solving time for individual routing instances not only enhances overall assay efficiency but also enables real-time adaptation to dynamic changes, such as unexpected errors or electrode degradation. From this perspective, improving both routing performance and solving time in MEDA biochips is of significant practical importance.

Several studies have focused specifically on routing in MEDA biochips. In [24], a routing method that incorporates droplet deformation to avoid obstacles leverages the flexible shape manipulation capability of MEDA. In this method, when a droplet encounters an obstacle, it deforms to bypass it and then returns to its original shape. Although this study discusses a velocity model, it does not utilize it for routing time reduction.

In contrast, Ref. [32] presents a routing technique that applies a velocity variation model to shorten routing time. Rather than deforming droplets merely to avoid obstacles, this method takes advantage of different velocities depending on droplet shape and movement direction to reduce routing time. Similarly, Ref. [33] extends this approach to parallel routing of multiple droplets. Furthermore, Ref. [34] integrates droplet splitting operations with the velocity model—previously used only for shape morphing—achieving even shorter routing times. However, methods [32,33,34] are based on integer programming, which poses a significant challenge in terms of solving time when applied to large solution spaces such as those in MEDA biochips.

To address this issue, the present study proposes a novel routing approach that reduces solving time while maintaining the quality of the routing solution. The proposed method introduces constraints that guide droplets toward the target cell based on distance information. By narrowing the solution space in this way, the overall solving time is significantly reduced. In other words, the aim of this study is to minimize droplet routing time while reducing the solving time.

The structure of this paper is as follows. Section 2 presents the problem setting and formulation of the proposed method. Section 3 describes the simulation experiments based on the formulation and discusses the results. Section 4 provides the conclusion of the paper.

## 2. Proposed Target-Guided Routing

### 2.1. Problem Description

**Figure 3:**
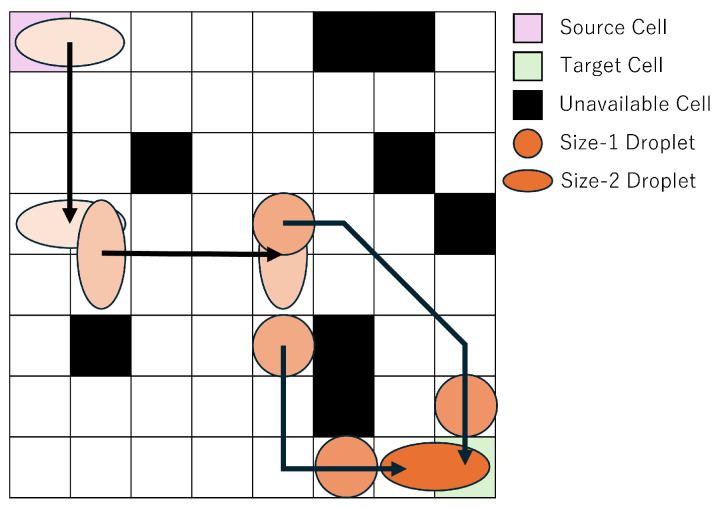
Routing Example.

In this study, we focus on droplet routing on MEDA biochips. MEDA biochips consist of a large number of regularly arranged cells, which can be represented as a grid-like diagram to simplify the discussion of droplet routing. Figure 3 and the following sections use simplified grid-based diagrams to explain the mathematical formulation of the routing problem. Figure 3 illustrates an example of droplet routing on a MEDA biochip. The top-left cell is the source cell, and the bottom-right cell is the target cell. For illustrative purposes, the passage of routing time is represented by the shading intensity of the droplet color. Routing refers to the operation of moving a droplet from the source cell to the target cell while avoiding unavailable cells, which serve as obstacles. The routing time is defined as the amount of time the droplet takes to move from the source cell to the target cell, and a shorter routing time leads to more efficient experimentation.

In this study, we consider one droplet with a size equivalent to two cells, where the size of a single cell is defined as one unit. A droplet of this size is referred to as a size-2 droplet, whereas a droplet occupying only one cell is referred to as a size-1 droplet. On a MEDA biochip, droplets can move in any of the eight surrounding directions, and can also morph, split, or merge at any time. As shown in Figure 3, the objective is to enable the droplet to reach the target cell in less time by utilizing shape morphing and splitting operations. In droplet splitting, droplets must be separated by at least one cell. In other words, to maintain a droplet as size-1, no other droplet can exist within the eight surrounding cells. Conversely, to merge droplets, one droplet must move into one of the eight neighboring cells of the other droplet. In this study, we assume that the temporal overhead for both splitting and merging is equivalent to the time required for a size-1 droplet to move one cell. Accordingly, we treat the required time for both operations as 1 in the experiments.

**Figure 4:**
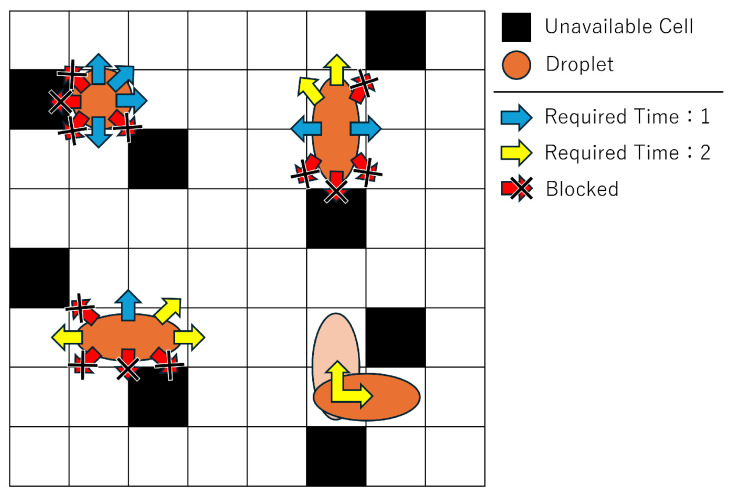
Direction and velocity of droplet movement.

Figure 4 illustrates the velocity variations depending on the droplet’s shape and movement direction, as well as the movement restrictions around unavailable cells. A distinctive feature of MEDA biochips is that the droplet’s movement speed depends on its shape and size. When moving in the direction of the blue arrows in Figure 4, 1 unit of time is required, while moving in the direction of the yellow arrows requires 2 units of time. These values may vary depending on the device and environment, but in this study, we adopt values based on the literature [32].

In addition, droplet movement directions are restricted near unavailable cells. An unavailable cell refers to a cell that cannot be used due to malfunction or droplet residue from repeated experiments. During operation, droplets must avoid such cells. Since droplets are driven by forces from the cells, movement directions are constrained near unavailable cells. Specifically, a droplet cannot move into or diagonally across an unavailable cell.

### 2.2. Routing Example

This section explains the proposed method using a concrete example. As shown in Figure 3, droplet routing on a MEDA biochip typically involves navigating from a source cell to a target cell while avoiding unavailable cells. There exist countless routing paths, and identifying a more efficient one contributes to accelerating the overall bioassay process. Previous studies [32,33,34] have successfully shortened routing time by utilizing shape-dependent droplet velocity, deformation, and splitting operations. However, a significant challenge remains in the long solving time required to discover such optimal solutions.

**Figure 5:**
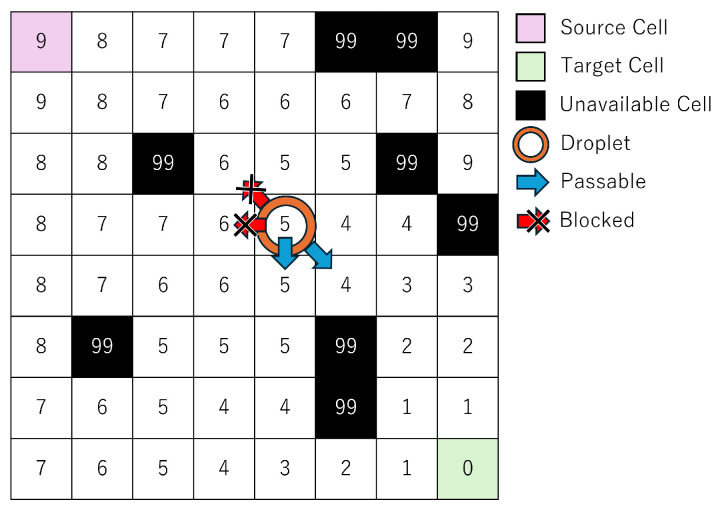
Example of distance information used in the proposed method.

To address this issue, the present study proposes a method that leverages distance information to the target cell, as illustrated in Figure 5.

At the start of routing, the target cell is set with a distance of zero, and the distance map is populated with the shortest travel time from each cell to the target cell. In other words, this distance information represents the minimum number of time steps required for a size-1 droplet located at any cell to reach the target. A key constraint, detailed in Section 2.3, is introduced for size-1 droplets: at time step *t*, a droplet may move only to a cell whose distance value is either equal to or exactly one less than that of the previous time step t−1. This constraint, as shown in Figure 5, limits the direction of droplet movement, reduces the solution space, and thereby shortens solving time. Furthermore, because the distance information guarantees the shortest path for size-1 droplets, solution quality is preserved.

In this study, we define solution quality as the total routing time—the number of time steps required for a droplet to reach its target cell. A shorter routing time indicates higher-quality routing. Solving time, in contrast, refers to the computational time taken to derive this routing solution using optimization. The constraint based on distance information improves solution quality by guiding droplets along optimal paths, while simultaneously reducing solving time by narrowing the feasible search space.

For size-2 droplets, however, factors such as shape, movement direction, and unavailable cells make routing more complex. Therefore, no such constraint is applied, and routing is solved using integer linear programming (ILP).

### 2.3. Formulation

This chapter formulates the proposed method. Table 1 presents the constants and variables used in the formulation.

In this study, we assume that the maximum droplet size is 2. Two droplets of size-1 are referred to as droplet A and droplet B, respectively. When droplets A and B are positioned in adjacent cells, they are treated as a single droplet of size-2. Each droplet is allowed to take one action per time step t. The actions of each droplet are managed using the values $direction_{a(b),t}$. The possible direction values and their corresponding actions are listed in Table 2.

Formula (1) represents the objective function, which aims to minimize the routing time of the droplets. The routing time is calculated by summing the time required for each *i*-th action of the droplets from the start to the end of the routing process and taking the longer of the two total times.

(1) minimize:routing_time

Formula (2) specifies the constraint for the routing termination condition. It checks whether the droplet’s position and shape are correct. To prevent the droplet from moving after routing is completed, the constraint ensures that the correct position and shape occur exactly once during the routing process.

(2) $$\left\{\sum_{t=0}^{T_{max}}\begin{array}{c} \left(x_{a,t}+x_{b,t}+w_{t}=X_{T}\times 2+1\right)\\ \wedge (y_{a,t}+y_{b,t}+h_{t}=Y_{T}\times 2+1)\\ \wedge \left(w_{t}=W_{T}\right)\wedge \left(h_{t}=H_{T}\right) \end{array}\right\}=1$$

Formula (3) defines the constraint on the droplet’s shape and size. This constraint ensures that a droplet always maintains one of the following forms: size-1, size-2 in a horizontal shape, or size-2 in a vertical shape.

(3) $$\forall t,\;\begin{array}{c} \left\{\left(w_{t}=1\right)\wedge \left(h_{t}=1\right)\right\}\vee \left\{\left(w_{t}=1\right)\wedge \left(h_{t}=2\right)\right\}\vee \left\{\left(w_{t}=2\right)\wedge \left(h_{t}=1\right)\right\} \end{array}$$

Formula (4) sets a constraint to ensure that the droplet remains within the chip boundaries. Specifically, the droplet’s position must always lie within the limits defined by the chip’s maximum width and height, and its coordinates must be no less than 1.

(4) $$\forall t,\;\begin{array}{c} \left(x_{a,t},x_{b,t}\leq W\right)\wedge \left(y_{a,t},y_{b,t}\leq H\right)\wedge \left(x_{a,t},x_{b,t},y_{a,t},y_{b,t}\geq 1\right) \end{array}$$

Formula (5) defines a constraint to avoid overlap between size-1 droplets. It guarantees that droplets A and B do not share the same location at any time *t*.

(5) $$\begin{array}{c} \forall t,\;\neg \left\{\left(x_{a,t}=x_{b,t}\right)\wedge \left(y_{a,t}=y_{b,t}\right)\right\} \end{array}$$

**Figure 6:**
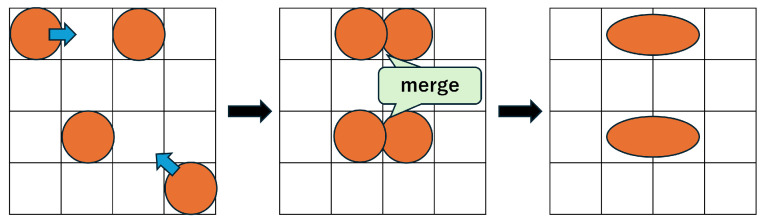
Droplet merging example.

Formulas (6)–(8) define constraints on droplet shapes and their positional relationships. For instance, when droplets are positioned side by side horizontally, droplets A and B are regarded as adjacent if their x-coordinates differ by 1 and their y-coordinates are identical. This situation represents a size-2 droplet and is described by Formulas (6) and (7). If a size-1 droplet moves to any of the 8 neighboring cells of another droplet, the two droplets may attract and merge, as shown in Figure 6 [35,36]. To prevent this and preserve droplet size, other droplets must be excluded from the 8 surrounding cells of a size-1 droplet. Specifically, Formula (8) enforces that either the x-coordinate or y-coordinate difference must be at least 2.

(6) $$\begin{array}{c} \forall t,\;\left\{\left(w_{t}=2\right)\wedge \left(h_{t}=1\right)\right\}\Rightarrow \left\{\left(\right|x_{a,t}-x_{b,t}\left|\;=1\right)\wedge \left(y_{a,t}=y_{b,t}\right)\right\} \end{array}$$

(7) $$\begin{array}{c} \forall t,\;\left\{\left(w_{t}=1\right)\wedge \left(h_{t}=2\right)\right\}\Rightarrow \left\{\left(\right|y_{a,t}-y_{b,t}\left|\;=1\right)\wedge \left(x_{a,t}=x_{b,t}\right)\right\} \end{array}$$

(8) $$\begin{array}{c} \forall t,\;\left\{\left(w_{t}=1\right)\wedge \left(h_{t}=1\right)\right\}\Rightarrow \left\{\left(\right|x_{a,t}-x_{b,t}\left|\;\geq 2)\vee (\right|y_{a,t}-y_{b,t}\left|\;\geq 2\right)\right\} \end{array}$$

The behavior of each droplet at time *t* is determined by the value of direction, which is defined in Table 2.

Formula (9) defines the behavioral constraint for droplets when the value of direction is 0. In this case, the droplet remains stationary.

(9) $$\forall t,\;\begin{array}{c} \left(direction_{a(b),t}=0\right)\Rightarrow \left\{\left(x_{a(b),t}=x_{a(b),t-1}\right)\wedge \left(y_{a(b),t}=y_{a(b),t-1}\right)\right\} \end{array}$$

Formula (10) defines the behavioral constraint for droplets when the value of direction is 1. In this case, the droplet moves one cell in the x-direction, regardless of whether the movement is to the left or right.

(10) $$\forall t,\;\begin{array}{c} \left(direction_{a(b),t}=1\right)\Rightarrow \left\{\left(\right|x_{a(b),t}-x_{a(b),t-1}\left|\;=1\right)\wedge \left(y_{a(b),t}=y_{a(b),t-1}\right)\right\} \end{array}$$

Formula (11) defines the behavioral constraint for droplets when the value of direction is 2. Similarly, in this case, the droplet moves one cell in the y-direction, regardless of whether the movement is upward or downward.

(11) $$\forall t,\;\begin{array}{c} \left(direction_{a(b),t}=2\right)\Rightarrow \left\{\left(x_{a(b),t}=x_{a(b),t-1}\right)\wedge \left(\right|y_{a(b),t}-y_{a(b),t-1}\left|\;=1\right)\right\} \end{array}$$

Formula (12) defines the behavioral constraint for droplets when the value of direction is 3. This constraint allows the droplet to move one cell diagonally, in any diagonal direction.

(12) $$\forall t,\;\begin{array}{c} \left(direction_{a(b),t}=3\right)\Rightarrow \left\{\left(\right|x_{a(b),t}-x_{a(b),t-1}\left|\;=1)\wedge (\right|y_{a(b),t}-y_{a(b),t-1}\left|\;=1\right)\right\} \end{array}$$

**Figure 7:**
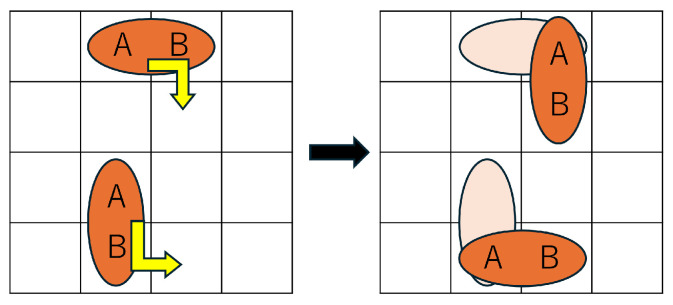
Droplet morphing example.

Formula (13) defines the behavioral constraint for droplets when the value of direction is 4. This constraint enables the droplet to perform a morphing action. Specifically, as shown in Figure 7, the shape (aspect ratio) of the droplet changes compared to the previous time step, and either droplet A or droplet B moves to the position previously occupied by the other, thereby realizing a shape transformation of a size-2 droplet.

(13) $$\forall t,\;\begin{array}{cc} \; & \{\left(direction_{a,t}=4\right)\wedge \left(direction_{b,t}=4\right)\}\Rightarrow \\ \; & \{\left(w_{t}\neq w_{t-1}\right)\wedge \left(h_{t}\neq h_{t-1}\right)\}\wedge \\ \; & \left\{\left(\left(x_{a,t}=x_{b,t-1}\right)\wedge \left(y_{a,t}=y_{b,t-1}\right)\right)\vee \left(\left(x_{b,t}=x_{a,t-1}\right)\wedge \left(y_{b,t}=y_{a,t-1}\right)\right)\right\} \end{array}$$

**Figure 8:**
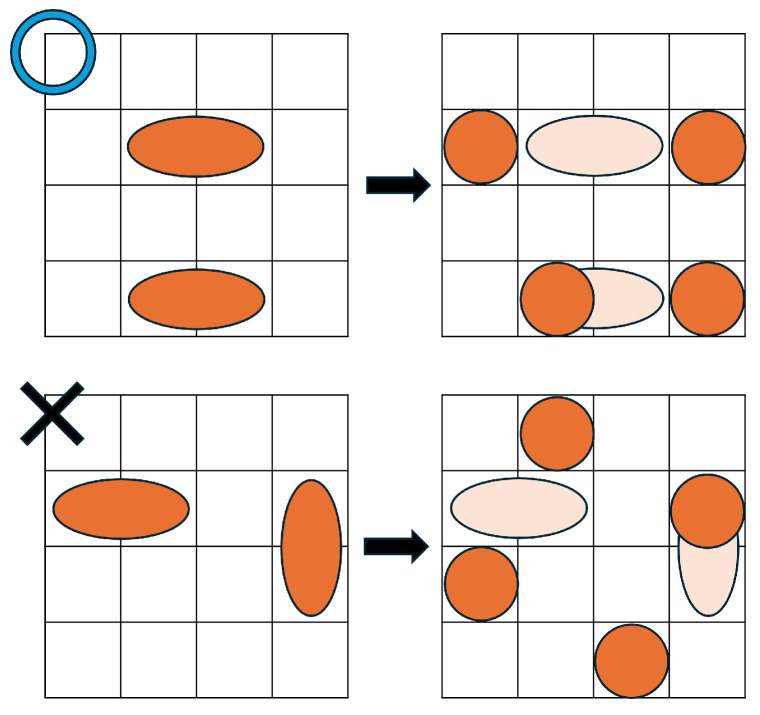
Droplet splitting example.

Formula (14) defines the constraints for droplet splitting. The splitting constraints used in the existing method [34] include certain patterns that are inconsistent with the physical behavior of droplets [37,38,39]. To resolve this issue, Formula (14) restricts splitting to physically plausible patterns. Specifically, as shown in Figure 8, a horizontally elongated size-2 droplet can only split in the horizontal direction. Splitting in the vertical or diagonal directions is not allowed.

(14) $$\forall t,\;\begin{array}{cc} \; & \{\left(direction_{a,t}=5\right)\wedge \left(direction_{b,t}=5\right)\}\Rightarrow \\ \; & \left\{\begin{array}{c} \left(\left(w_{t}=1\right)\wedge \left(h_{t}=1\right)\right)\wedge \left(\left(w_{t-1}=2\right)\vee \left(h_{t-1}=2\right)\right) \end{array}\right\}\wedge \\ \; & \left[\begin{array}{c} \left\{(\right|x_{a,t}-x_{a,t-1}\left|\;=1\right)\wedge \left(y_{a,t}=y_{a,t-1}\right)\\ \wedge \left(\right|x_{b,t}-x_{b,t-1}\left|\;\leq 1\right)\wedge \left(y_{b,t}=y_{b,t-1}\right)\}\\ +\left\{(\right|x_{b,t}-x_{b,t-1}\left|\;=1\right)\wedge \left(y_{b,t}=y_{b,t-1}\right)\\ \wedge \left(\right|x_{a,t}-x_{a,t-1}\left|\;\leq 1\right)\wedge \left(y_{a,t}=y_{a,t-1}\right)\}\\ +\left\{(\right|y_{a,t}-y_{a,t-1}\left|\;=1\right)\wedge \left(x_{a,t}=x_{a,t-1}\right)\\ \wedge \left(\right|y_{b,t}-y_{b,t-1}\left|\;\leq 1\right)\wedge \left(x_{b,t}=x_{b,t-1}\right)\}\\ +\left\{(\right|y_{b,t}-y_{b,t-1}\left|\;=1\right)\wedge \left(x_{b,t}=x_{b,t-1}\right)\\ \wedge \left(\right|y_{a,t}-y_{a,t-1}\left|\;\leq 1\right)\wedge \left(x_{a,t}=x_{a,t-1}\right)\} \end{array}\right]=1 \end{array}$$

Formulas (15)–(19) define constraints concerning droplet movement near unavailable cells. In these formulas, $\left(X_{u,i},Y_{u,i}\right)$ denotes the coordinates of the *i*-th unavailable cell. Droplets are prohibited from occupying these unavailable cells, and their movement is restricted in the vicinity. Formula (15) prohibits the droplet from entering unavailable cells by ensuring that the droplet’s coordinates do not coincide with those of any unavailable cell.

(15) $$\forall t,i,\;\neg \left\{\begin{array}{c} \left(\left(x_{a,t}=X_{u,i}\right)\wedge \left(y_{a,t}=Y_{u,i}\right)\right)\vee \left(\left(x_{b,t}=X_{u,i}\right)\wedge \left(y_{b,t}=Y_{u,i}\right)\right) \end{array}\right\}$$

Formulas (16)–(19) define the constraints that restrict diagonal movements of droplets near unavailable cells. Notably, Formula (16) addresses cases where an unavailable cell lies to the left of the droplet. Additional constraints, as defined in Formulas (17)–(19), handle cases where unavailable cells are located to the right, above, or below the droplet.

(16) $$\begin{array}{c} \forall t,i,\;\left\{\left(x_{a(b),t-1}-X_{u,i}=1\right)\wedge \left(y_{a(b),t-1}=Y_{u,i}\right)\right\}\Rightarrow \left(x_{a(b),t}\neq x_{a(b),t-1}-1\right) \end{array}$$

(17) $$\begin{array}{c} \forall t,i,\;\left\{\left(Y_{u,i}-y_{a(b),t-1}=1\right)\wedge \left(x_{a(b),t-1}=X_{u,i}\right)\right\}\Rightarrow \left(y_{a(b),t}\neq y_{a(b),t-1}+1\right) \end{array}$$

(18) $$\begin{array}{c} \forall t,i,\;\left\{\left(X_{u,i}-x_{a(b),t-1}=1\right)\wedge \left(y_{a(b),t-1}=Y_{u,i}\right)\right\}\Rightarrow \left(x_{a(b),t}\neq x_{a(b),t-1}+1\right) \end{array}$$

(19) $$\begin{array}{c} \forall t,i,\;\left\{\left(y_{a(b),t-1}-Y_{u,i}=1\right)\wedge \left(x_{a(b),t-1}=X_{u,i}\right)\right\}\Rightarrow \left(y_{a(b),t}\neq y_{a(b),t-1}-1\right) \end{array}$$

Formula (20) defines the constraint proposed in this study to guide droplets toward the target cell. At the beginning of the routing process, the distance from each cell to the target cell is set to 0 at the target and increases with distance. This information is used to restrict the droplet’s movement so that it approaches the target. Specifically, for a size-1 droplet, the difference in the value of $Dmap_{y,x}$ between time steps t−1 and *t* must be either 0 or 1. By eliminating movement choices that lead away from the target, this constraint helps reduce solving time.

(20) $$\forall t,\;\begin{array}{cc} \; & \{\left(w_{t}=1\right)\wedge \left(h_{t}=1\right)\}\Rightarrow \\ \; & \left\{\begin{array}{c} \left(\left(Dmap_{y_{a,t-1},x_{a,t-1}}-Dmap_{y_{a,t},x_{a,t}}\right)\in \left\{0,1\right\}\right)\wedge \\ \left(\left(Dmap_{y_{b,t-1},x_{b,t-1}}-Dmap_{y_{b,t},x_{b,t}}\right)\in \left\{0,1\right\}\right) \end{array}\right\} \end{array}$$

Constraint (21) enforces the droplet to perform a splitting operation at time step t=1. As demonstrated in [34], considering a velocity model dependent on droplet shape, size-1 droplets can follow paths with greater flexibility compared to size-2 droplets, potentially leading to improved solution quality. Additionally, constraint (20) ensures that the size-1 droplet follows the shortest path. Therefore, by forcing an initial split into size-1 droplets, it is expected that the method can both guarantee solution quality and reduce solving time. This constraint is not used in the standard proposed method but is implemented only in the additional variation referred to as “proposed + t1 con.”

(21) $$\begin{array}{c} \left(direction_{a,1}=5\right)\wedge \left(direction_{b,1}=5\right) \end{array}$$

## 3. Experiments

### 3.1. Setup

In this study, the objective is to optimize the total routing time of droplets in the droplet routing process. Routing is considered complete when a droplet moves from the source cell to the target cell. To evaluate the fundamental effectiveness of the proposed method, the experiments were conducted in simulation, and only a single size-2 droplet or two size-1 droplets after splitting were considered. At any time step, a droplet can move to any of the eight surrounding cells, morph, split, or merge. Experiments were conducted assuming MEDA biochips with chip sizes of 8 × 8, 10 × 10, and 12 × 12. Unavailable cells were randomly placed at rates of 10%, 20%, and 30%, with 10 trials conducted for each setting (except for the 12 × 12 chip with 30% invalid cells, which was tested 20 times). The experimental results were compared across three methods: the existing method [34], the proposed method, and a variation of the proposed method that includes a constraint forcing droplet splitting at time step 1 (proposed + t1 con.).

The existing method aims to reduce droplet routing time by considering shape-dependent velocity changes and utilizing droplet morphing and splitting operations. The proposed method applies distance-map-based constraints to each of the split size-1 droplets individually, guiding them along the shortest path to the target cell. This approach aims to minimize droplet routing time while also reducing solving time. Based on the characteristics of this method, it is assumed that the fastest routing is achieved when the droplet is always split at the initial stage and each size-1 droplet is independently guided via its shortest path. This assumption is experimentally evaluated as the proposed + t1 con. method.

Each method corresponds to the following set of constraints:

- Existing method [34]: from constraint (1) to constraint (19)
- Proposed method: from constraint (1) to constraint (20)
- Proposed + t1 con. method: from constraint (1) to constraint (21)

Table 3 shows the initial values of the constants. These values are determined for each problem and provided as inputs during the experiments. The initial position and shape of the droplet at the start of routing, the coordinates of the target cell, and the final shape of the droplet at the end of routing are fixed across all problems according to the values shown in the table.

All experiments were carried out using the mathematical optimization solver ILOG CPLEX Optimization Studio 22.1.0. The computational environment consisted of an Intel Core i7-10700 processor and 64 GB of RAM. A time limit of 50,000 s (CPU time) was imposed, and if the optimal solution was not found within this time, the best solution obtained within the time limit was adopted as the result. If no result is obtained within the time limit, the graph is not shown.

### 3.2. Results

**Figure 9:**
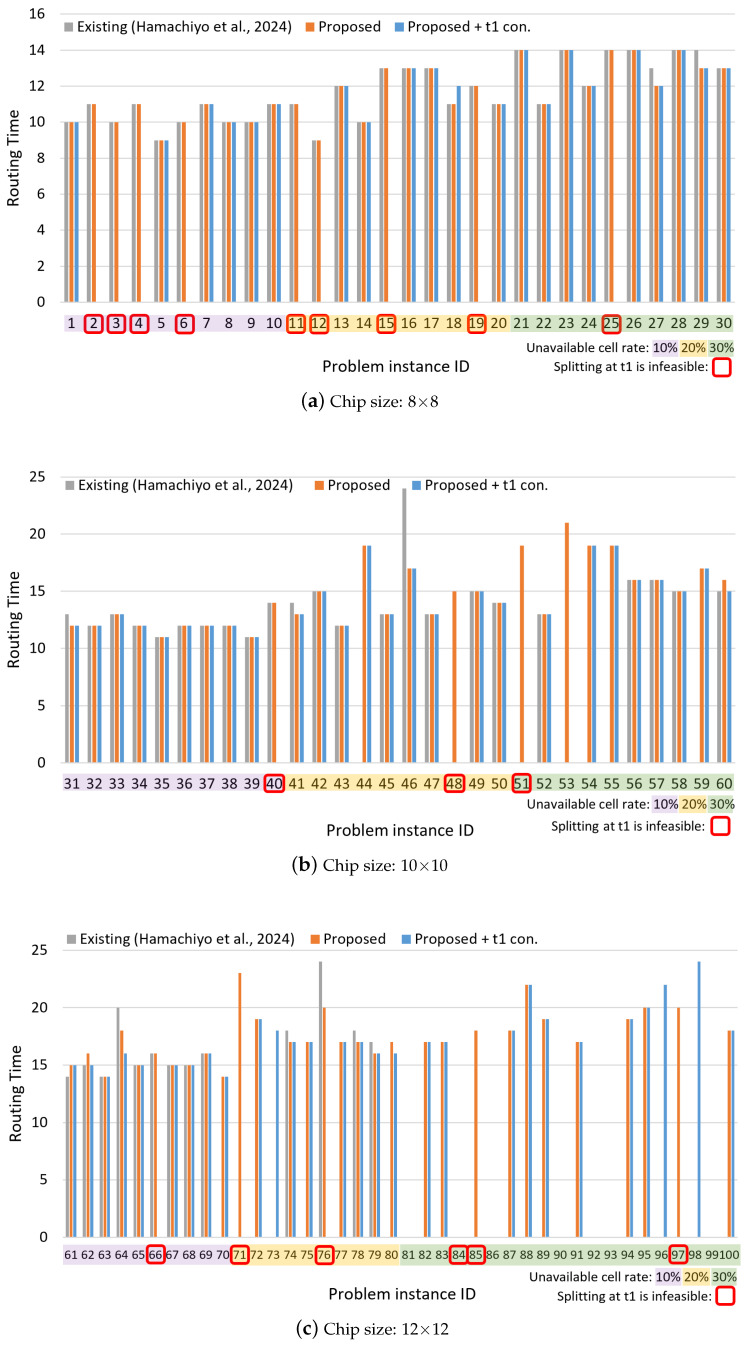
Routing time results for different chip sizes: (**a**) 8 × 8, (**b**) 10 × 10, and (**c**) 12 × 12. Each bar chart shows the results grouped by the ratio of unavailable cells, represented by different colors. Cases where droplet splitting at time step 1 is impossible due to the chip layout are indicated with red rectangles [34].

**Figure 10:**
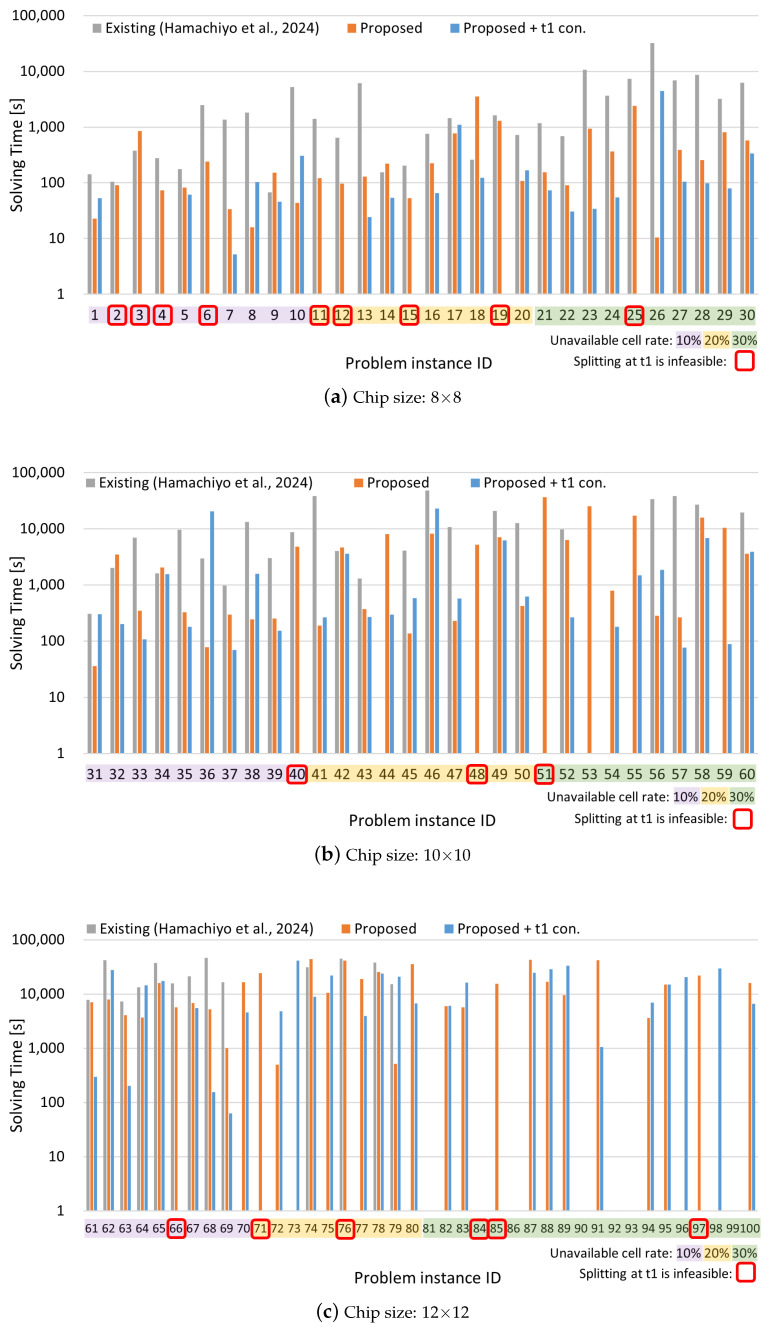
Solving time results for different chip sizes: (**a**) 8 × 8, (**b**) 10 × 10, and (**c**) 12 × 12. Each bar chart is color-coded according to the ratio of unavailable cells. The vertical axis uses a logarithmic scale. Cases in which droplet splitting is impossible at time step 1 due to chip layout constraints are highlighted with red rectangles [34].

The experimental results are presented in Figure 9 and Figure 10. The horizontal axis of each graph represents the problem instance ID, while the vertical axes indicate the routing time, which is the total movement time of droplets, and the solving time, which is the time required to find the solution. The problem instance IDs are color-coded according to the ratio of invalid cells: purple for 10%, yellow for 20%, and green for 30%. The problem instances with the following IDs, which are highlighted in red on the graph, could not be solved by the Proposed + t1 constraint method due to the placement of unavailable cells on the board, which makes droplet splitting at time step 1 infeasible: 2, 3, 4, 6, 11, 12, 15, 19, 25, 40, 48, 51, 66, 71, 76, 84, 85, 97.

From Figure 9, it can be observed that the proposed method achieves routing times comparable to those of the existing method in most cases. Furthermore, for certain problem instances—particularly those involving larger chip sizes and higher rates of unavailable cells—the existing method fails to find solutions, whereas the proposed method succeeds. Figure 10 shows that the proposed method also significantly reduces solving time for many instances compared to the existing method, with some cases achieving reductions of over 99%. Even in instances where the existing method finds a solution more quickly, the proposed method often yields solutions with shorter routing times, indicating superior solution quality. However, in a few of these instances, the existing method was able to find a solution of comparable quality in a shorter amount of time.

Table 4 presents the average results for each chip size and obstacle-cell ratio, calculated across 10 instances per case, along with the overall average across all problem instances. Regarding routing time, the baseline method failed to find solutions for the 12 × 12 chip with 30% unavailable cells, and thus, those instances are excluded from the average, which likely results in a deceptively lower value. For solving time, the proposed method outperforms the existing method in nearly all cases. The average reduction rate (excluding the unsolvable 12 × 12–30% cases) achieved by the proposed method is 67.5%, and even when considering all cases, a 37.7% improvement is observed. Furthermore, the enhanced version of the proposed method, which includes a constraint to enforce droplet splitting at time step 1, achieved 74.5% average improvement (excluding the 12 × 12–30% cases), and a 45.3% overall improvement.

Some problem instances, due to the nature of the board layout, do not allow droplet splitting at time step 1. Table 5 shows the results excluding those specific instances. Even in this subset, the proposed method outperforms the existing method, achieving a 70.9% average reduction (excluding the 12 × 12–30% cases) and a 46.9% overall improvement. With the additional constraint for splitting at time step 1, the method achieves a 73.6% average reduction and a 49.7% overall improvement, respectively. In other words, in terms of overall average, the extended method with the time step 1 splitting constraint achieved approximately 5.4% shorter solving time compared to the proposed method. In terms of solution success rate, the proposed method also shows substantial improvement—90 out of 100 problems were solved successfully, compared to 66 out of 100 with the baseline method.

## 4. Conclusions

This paper proposed an acceleration method for droplet routing on MEDA biochips, taking into account shape-dependent velocity variations. By incorporating a constraint that encourages droplets to move closer to their target cells based on a distance map, the method successfully reduced solving time without degrading solution quality. Experimental results showed that the proposed method achieved approximately a 67.5% reduction in solving time compared to existing approaches. Furthermore, the extended version that incorporates a constraint to enforce droplet splitting at time step 1 achieved an additional average reduction of approximately 5.4% in solving time compared to the proposed method, without affecting solution quality. However, this extended method cannot handle chip layouts where splitting at time step 1 is not possible. Therefore, selecting between the two methods based on the layout is considered effective. As future work, since a time limit was imposed during experiments and optimality could not be guaranteed, we plan to investigate methods that can reduce solving time while ensuring optimality.

## Figures and Tables

**Table 1:** Notations.

Character	Meaning
routing_time	Time droplets to move from source to target
$T_{max}$	Upper bound of the time step *t*
W,H	Biochips width and height
$x_{a(b),t},y_{a(b),t}$	Position of droplet A(B) at time *t*
$w_{t},h_{t}$	Droplet shape at time *t*
$direction_{a(b),t}$	Droplet operation number at time *t*
$X_{u,i},Y_{u,i}$	Position of the *i*-th unavailable cell
$W_{T},H_{T}$	Droplet shape at the target cell
$X_{T},Y_{T}$	Coordinates of the target cell
$Dmap_{y,x}$	Distance map to target cell

**Table 2:** Meanings of Directions.

Direction	Droplet Behavior
0	Stay in place
1	Move one cell in x-direction
2	Move one cell in y-direction
3	Move one cell diagonally
4	Morph
5	Split

**Table 3:** Inputs.

Character	Initial Value	Meaning
W,H	Problem-dependent	Biochips width and height
$T_{max}$	W+H	Upper bound of the time step *t*
$W_{S},H_{S}$	$W_{S}=2,H_{S}=1$	Droplet shape at the start of routing
$W_{T},H_{T}$	$W_{T}=2,H_{T}=1$	Droplet shape at the target cell
$X_{S,a(b)},Y_{S,a(b)}$	A(1, 1), B(2, 1)	Coordinates of the droplet at the start
$X_{T},Y_{T}$	(W, H)	Coordinates of the target cell
$X_{u,i},Y_{u,i}$	Problem-dependent	Position of the *i*-th unavailable cell
$Dmap_{y,x}$	Problem-dependent	Distance map to target cell

**Table 4:** Average (all).

		Existing [34]		Proposed		Proposed + t1 con.	
Size	Rate	Rou-Time	Sol-Time [s]	Rou-Time	Sol-Time [s]	Rou-Time	Sol-Time [s]
	10%	10.3	1204.1	10.3	161.1	10.2	95.8
8 × 8	20%	11.5	1341.7	11.5	658.2	11.8	255.6
	30%	13.3	8102.2	13.1	597.6	13.0	587.1
	10%	12.2	4950.3	12.1	1192.4	11.9	2734.4
10 × 10	20%	15.0	17,518.9	14.6	3439.6	14.6	3918.7
	30%	15.0	25,669.9	17.1	11,694.6	16.3	1839.2
	10%	15.6	23,109.5	15.4	7388.5	15.0	7827.0
12 × 12	20%	19.3	32,265.4	18.1	22,366.5	17.1	16,520.4
	30%	N/A	N/A	18.6	17,692.7	19.4	17,137.9
	All	13.4	11,538.3	14.5	7191.5	14.7	6308.1

**Table 5:** Average (excluding constraint-unsatisfiable instances).

		Existing [34]		Proposed		Proposed + t1 con.	
Size	Rate	Rou-Time	Sol-Time [s]	Rou-Time	Sol-Time [s]	Rou-Time	Sol-Time [s]
	10%	10.2	1468.1	10.2	58.3	10.2	95.8
8 × 8	20%	11.7	1588.9	11.7	834.0	11.8	255.6
	30%	13.2	8181.9	13.0	398.9	13.0	587.1
	10%	12.0	4527.7	11.9	786.7	11.9	2734.4
10 × 10	20%	15.0	17,518.9	14.6	3243.0	14.6	3918.7
	30%	15.0	25,669.9	16.9	8903.8	16.3	1839.2
	10%	15.5	24,026.5	15.3	7573.1	15.0	7827.0
12 ×12	20%	17.7	28,030.8	17.1	19,421.4	17.1	16,520.4
	30%	N/A	N/A	18.6	17,467.5	19.4	17,137.9
	All	13.5	12,546.9	14.6	6666.6	14.7	6308.1

## Data Availability

The original contributions presented in this study are included in the article. Further inquiries can be directed to the corresponding author.
